# Supplementary material for: Synapsin-Promoted Caveolin-1 Overexpression Maintains Mitochondrial Morphology and Function in PSAPP Alzheimer’s Disease Mice
Source: Cells. 2021 Sep 20;10(9):2487. doi: 10.3390/cells10092487 (PMC8467690; doi:10.3390/cells10092487)
Supplement: Supplementary file 1 [file cells-10-02487-s001.zip › cells-1307087-supplementary.pdf]

cells

MDPI

**Supplemental Figures**

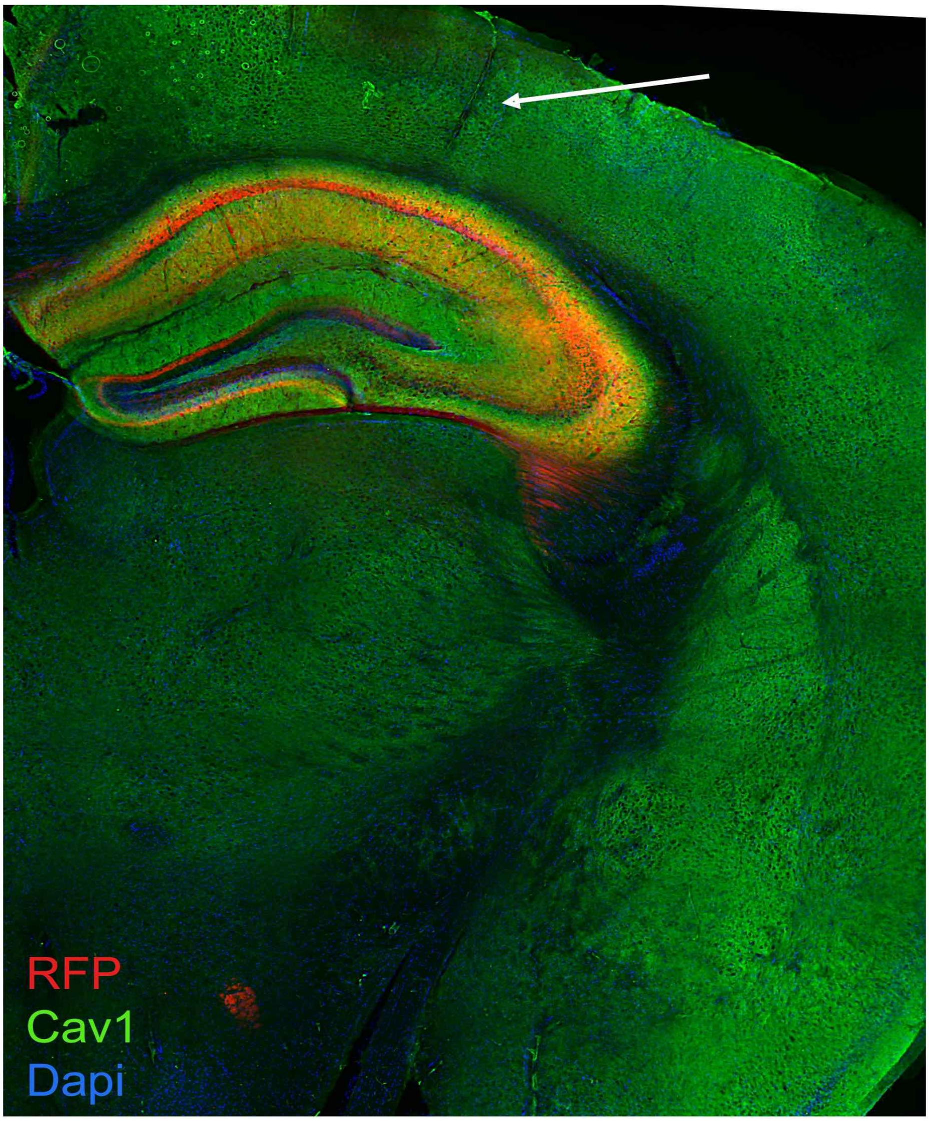


**Supplement Figure S1. Representative image of stereo-microinjection of WT-*SynRFP* at 12m age.** Arrow indicates needle track of original injection site.

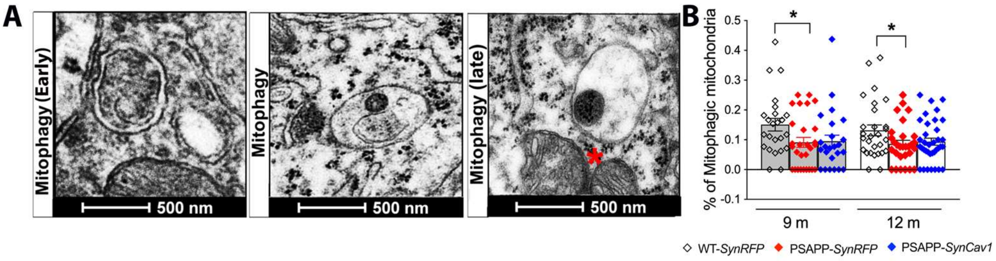


**Supplement Figure S2. Analysis of mitophagy via EM. (A) Representative images of mitophagy. (B) Quantification of mitophagy event at both 9 and 12 months of age.** Both PSAPP-SynRFP (p=0.03 at 9 and 12 months) and PSAPP-SynCav1((p=0.051 at 9 months, p=0.059 at 12 months) group showed lower mitophagy activity compared to WT, no significant change was found between two PSAPP groups. Data are presented as mean ± S.E.M., n = 5-6 per group. Data were analyzed using one-way analysis of variance (ANOVA) followed by Tukey multiple comparisons test. Significance was assumed when $p < 0.05$. *$p < 0.05$.

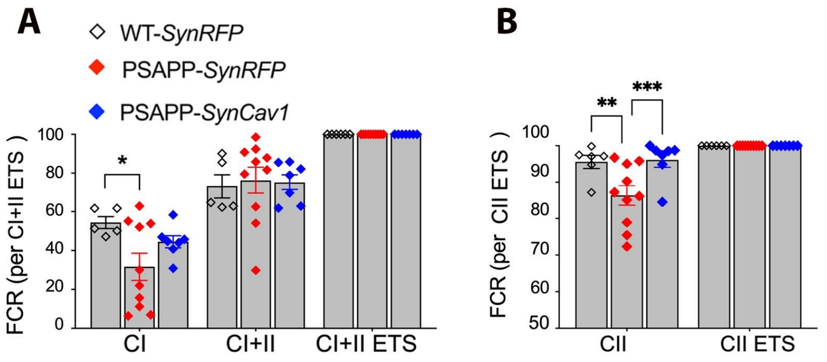


**Supplement Figure S3. (A-B)** Quantification of FCR from hippocampal homogenates at 12 months of age. PSAPP-*SynRFP* showed significant reduction in both CI and CII FCR normalized to maximal ETS compared to WT-*SynRFP*, PSAPP-*SynCav1* showed higher FCR in CII FCR compared to PSAPP-*SynRFP*, and a no significant increase trend in CI FCR compared to PSAPP-*SynRFP*. No significant change was found between WT-*SynRFP* and PSAPP- *SynCav1*. Data are presented as mean ± S.E.M., n = 5-6 per group. Data were analyzed using one-way analysis of variance (ANOVA) followed by Tukey multiple comparisons test. Significance was assumed when $p < 0.05$. *$p < 0.05$, **$p < 0.01$, ***$p < 0.001$.
